# Supplementary material for: From sequence to enzyme mechanism using multi-label machine learning
Source: BMC Bioinformatics. 2014 May 19;15:150. doi: 10.1186/1471-2105-15-150 (PMC4229970; doi:10.1186/1471-2105-15-150)
Supplement: Additional file 2 — Java code of ml2db. Additional file ml2db_code.tar.gz contains the Java source code to run the multi-label machine learning experiments and save the results to database. The code’s Javadoc is included. [file 1471-2105-15-150-S2.zip › additional file 2/ml2db/ml2db/doc/index-files/index-3.html]

C-Index


JavaScript is disabled on your browser.


- Overview
- Package
- Class
- Use
- Tree
- Deprecated
- Index
- Help

- Prev Letter
- Next Letter

- Frames
- No Frames

- All Classes

A B C D E F G H I J K L M N O P Q R S T U V W X 


## C

CA\_CLASS1 - Static variable in class test.ml2db.dataharness.DataOne


CA\_CLASS2 - Static variable in class test.ml2db.dataharness.DataOne


CA\_CLASS3 - Static variable in class test.ml2db.dataharness.DataOne


CA\_CLASS4 - Static variable in class test.ml2db.dataharness.DataOne


calculateAllDistances() - Method in class main.ml2db.al.utility.globaldistance.DistancesCalculator
:   Fill storage with all possible distances (before serialization)

calculateCoocurrence(MultiLabelInstances) - Method in class main.ml2db.mulan.mulanarff.Statistics
:   This method calculates and prints a matrix with the coocurrences of   
    pairs of labels

calculateFrequency() - Method in class main.ml2db.al.utility.pickers.byfrequency.ByFrequencyPicker
:   Orders the attribute set by descending frequency

calculatePhi(MultiLabelInstances) - Method in class main.ml2db.mulan.mulanarff.Statistics
:   Calculates phi correlation

calculateStats(MultiLabelInstances) - Method in class main.ml2db.mulan.mulanarff.Statistics
:   calculates various multilabel statistics, such as label cardinality,   
    label density and the set of distinct labels along with their frequency

calculateUtilities(int) - Method in class main.ml2db.al.ActiveLearning
:   Calculate utility metric for each instance.

cardinality() - Method in class main.ml2db.mulan.mulanarff.Statistics
:   returns the label cardinality of the dataset

checkAndGetFilePath(ArffRecordsTable, String, int) - Static method in class main.ml2db.mulan.mulanarff.database.MulanArffRecord
:   Returns the file path if the file is readable.

checkAndInitialise(String, DbManagers, boolean) - Method in class main.ml2db.weka.Arff


checkDouble(String, Double) - Method in class test.ml2db.mulan.learn.traintest.EvaluationMetricsTest


checkDouble(String, Double) - Method in class test.ml2db.mulan.learn.traintest.ResultCheckerTest


checkInt(String, Integer) - Method in class test.ml2db.mulan.learn.traintest.ResultCheckerTest


checkPropertiesKeys(Properties) - Method in class main.ml2db.weka.ArffProps


checkTestArffRecord() - Method in class main.ml2db.mulan.learn.traintest.TrainTestInputManager


checkTrainArffRecord() - Method in class main.ml2db.mulan.learn.crosseval.CrossInputManager


CLASS1 - Static variable in class test.ml2db.dataharness.DataOne


CLASS2 - Static variable in class test.ml2db.dataharness.DataOne


CLASS3 - Static variable in class test.ml2db.dataharness.DataOne


CLASS4 - Static variable in class test.ml2db.dataharness.DataOne


CLASS\_ATTRIBUTES\_TAG - Static variable in class main.ml2db.weka.InstAttValueMap


CLASS\_FIELD - Static variable in class test.ml2db.dataharness.DataTableTest


CLASS\_NAME\_PROP - Static variable in class main.ml2db.weka.ArffProps
:   The class name.

CLASS\_TO\_STRING - Static variable in class test.ml2db.dataharness.DataOne


classColumn() - Static method in class test.ml2db.dataharness.DataOne


classValues() - Static method in class test.ml2db.dataharness.DataOne
:   Results of a select distinct instance, class query

clone(Serializable) - Static method in class main.ml2db.Serializer
:   To test where in a class an instance of Object is created, hence making
    serialization impossible.

closeConnection() - Method in class main.ml2db.mulan.learn.traintest.TrainTestOutputManager


closeConnections() - Method in class main.ml2db.DbManagers
:   Closes all three database connections

colourLabelled(IdMultiLabelInstances) - Method in class main.ml2db.graphviz.GraphVizArffColoured
:   Assigns a colour depending on an integer (from 0 to >4)

colourPredictedInstances(TreeMap<IdInstance, Double>) - Method in class main.ml2db.graphviz.GraphVizArffColoured


compareTo(IdInstance) - Method in class main.ml2db.mulan.mulanarff.IdInstance
:   Order by instance identifier string

completeDotLine(String) - Method in class main.ml2db.graphviz.GraphVizArff


CONFIDENCE - Static variable in class main.ml2db.mulan.predict.PredictionsRecordTable
:   the confidence in the prediction, where available

ConfidenceCalculator - Class in main.ml2db.al.utility.confidence
:   Gets the confidence of a multi-label prediction and calculates how close it
    is to 50% (maximum uncertainty)

ConfidenceCalculator(int) - Constructor for class main.ml2db.al.utility.confidence.ConfidenceCalculator


ConfidenceCalculatorTest - Class in test.ml2db.al.utility.confidence


ConfidenceCalculatorTest() - Constructor for class test.ml2db.al.utility.confidence.ConfidenceCalculatorTest


ConfidenceMetric - Class in main.ml2db.al.utility.confidence
:   Combines a Positioning metric (a metric that considers positively if an
    instance is close to other unlabelled instances and distant from other
    labelled instances) with the average certainty of the instance prediction
    (that is, how distant the average of confidence over all labels is from 50%)

ConfidenceMetric() - Constructor for class main.ml2db.al.utility.confidence.ConfidenceMetric


ConfidenceMetricTest - Class in test.ml2db.al.utility.confidence


ConfidenceMetricTest() - Constructor for class test.ml2db.al.utility.confidence.ConfidenceMetricTest


ConfidencesStorage - Class in main.ml2db.al.utility.confidence


ConfidencesStorage() - Constructor for class main.ml2db.al.utility.confidence.ConfidencesStorage


ConfidencesStorage(int) - Constructor for class main.ml2db.al.utility.confidence.ConfidencesStorage


coreKeys() - Method in class main.ml2db.weka.ArffProps
:   The core ARFF properties, essential to load the data from a Weka ARFF
    file

coreKeys() - Method in class main.ml2db.weka.ArffPropsFromFile
:   The core ARFF properties, essential to generate a Weka ARFF file

corePropertiesAreCorrect(Properties) - Method in class main.ml2db.weka.ArffProps


corePropertiesAreCorrect(Properties) - Method in class main.ml2db.weka.ArffPropsFromFile


createAttributes() - Method in class main.ml2db.weka.AttributeFactory
:   Generate the set of attributes

createNewArff(int) - Method in class main.ml2db.mulan.attributesfiltered.AttFilter


createNewTestArff() - Method in class main.ml2db.mulan.attributesfiltered.AttFilter


createNewTrainArff() - Method in class main.ml2db.mulan.attributesfiltered.AttFilter


createRow(int, int, String, String, Evaluation, String, String, String, String, String, String, TableManager) - Method in class main.ml2db.al.database.AlEvaluationTable


createRow(int, int, String, MulanLearner, String, String, TableManager) - Method in class main.ml2db.al.database.AlExperimentsTable
:   Create a row with the experiment details

createRow(Object[], TableManager) - Method in class main.ml2db.al.database.AlExperimentsTable


createRow(int, int, String, String, double, String, TableManager) - Method in class main.ml2db.al.database.QueryTable


createRow(Object[], TableManager) - Method in class main.ml2db.al.database.QueryTable


createRow(int, int, String, double, TableManager) - Method in class main.ml2db.al.database.UtilityTable


createRow(Object[], TableManager) - Method in class main.ml2db.al.database.UtilityTable


createRow(Properties) - Method in class main.ml2db.weka.ArffRecordsTable


createTables() - Method in class main.ml2db.al.database.AlDbCreator


createTestTable(String) - Method in class test.ml2db.dataharness.DataTableTest


CrossEvaluator - Class in main.ml2db.mulan.learn.crosseval
:   Class created to execute cross-evaluations and extract individual evaluation
    results (not possible from the Mulan class Evaluator, because only averaged
    results are returned).

CrossEvaluator(CrossExperimenter, boolean) - Constructor for class main.ml2db.mulan.learn.crosseval.CrossEvaluator


CrossEvaluatorTest - Class in test.ml2db.mulan.learn
:   Class

CrossEvaluatorTest() - Constructor for class test.ml2db.mulan.learn.CrossEvaluatorTest


CrossExperimenter - Class in main.ml2db.mulan.learn.crosseval
:   Using Mulan (multi-label machine learning), this class initialises and
    executes either Train-Test evaluation (if two different arff records are
    given) (calling `MulanTrainTestEvaluator`) or cross-evaluations (calling
    `MulanCrossEvaluator`) if only one arff record is given.

CrossExperimenter(DbManagers, int, MulanLearner, String) - Constructor for class main.ml2db.mulan.learn.crosseval.CrossExperimenter


CrossExperimenter(IdMultiLabelInstances, MulanLearner, DbManagers, String) - Constructor for class main.ml2db.mulan.learn.crosseval.CrossExperimenter


CrossInputManager - Class in main.ml2db.mulan.learn.crosseval
:   An input manager for a single dataset (set of instances).

CrossInputManager(IdMultiLabelInstances, CrossExperimenter) - Constructor for class main.ml2db.mulan.learn.crosseval.CrossInputManager
:   Gets the instances as object.

CrossInputManager(int, DbManagers, CrossExperimenter) - Constructor for class main.ml2db.mulan.learn.crosseval.CrossInputManager
:   Gets the instances from database arff record.

CrossOutputManager - Class in main.ml2db.mulan.learn.crosseval
:   Abstract class to save the output (evaluation results) of a cross or
    train-test evaluation.

CrossOutputManager(DbManager, String, Table, CrossExperimenter) - Constructor for class main.ml2db.mulan.learn.crosseval.CrossOutputManager

A B C D E F G H I J K L M N O P Q R S T U V W X

- Overview
- Package
- Class
- Use
- Tree
- Deprecated
- Index
- Help

- Prev Letter
- Next Letter

- Frames
- No Frames

- All Classes
